# Osteogenesis and osteoclast inhibition in rheumatoid arthritis patients treated with bisphosphonates alone or in combination with pitavastatin over an 18-month follow-up after more than 4 years of treatment with bisphosphonates

**DOI:** 10.1186/ar4063

**Published:** 2012-10-18

**Authors:** Masakazu Nagashima, Hiroshi Takahashi, Kenichi Shimane, Yuichi Nagase, Koichi Wauke

**Affiliations:** 1 Department of Rheumatology, Tokyo Metropolitan Bokutoh Hospital, Kohtohbashi 4-23-15, Sumida-ku, Tokyo 130-8575, Japan; 2 Hospitality Care Garden Seisei Rehabilitation Hospital, Kasuga 2-12-25, Aoi-ku, Shizuoka 420-0823, Japan

## Abstract

**Introduction:**

To investigate the effects of bisphosphonates (Bis) (etidronate, alendronate, and risedronate), alone and in combination with statin, on the BMD (bone mineral density) and bone metabolism of rheumatoid arthritis (RA) patients.

**Methods:**

Seventy-seven RA patients who had been receiving prednisolone (PSL) and Bis for over 4 years were divided into two groups: Bis and Bis + statin (*n *= 42 and 35; average age, 66.4 and 65.3 years; average disease duration, 24.9 and 20.8 years; average PSL dose, 2.4 and 2.7 mg, respectively). Serum levels of NTX (N-terminal telopeptide of type I collagen), TRACP-5b (tartrate-resistant acid phosphate-5b), PICP (C-terminal propeptide of type I procollagen), and RANKL (receptor activator of NF-κB ligand) were measured over an 18-month period of treatment and follow-up. The BMD levels of the two groups at the radius, lumbar spine, and femoral neck were compared using DXA (dual-energy x-ray absorptiometry).

**Results:**

A significant increase was only observed in the BMD of the lumbar spine at 18-months, but the BMDs of the radius and femoral neck decreased during the follow-up period in the Bis group. Meanwhile, a significant increase was observed in the BMD of the lumbar spine in the Bis + statin group during administration and the BMDs of the radius and femoral neck stayed at baseline. Among the markers of bone metabolism, serum NTX was up-regulated after 6 months in the Bis + statin group. Serum TRACP-5b was significantly increased during the follow-up period in the Bis + statin group, but only at 18 months in the Bis group. Serum PICP recovered to base line in the Bis + statin group, whereas that in the Bis group did not observably recover during the post-administration follow-up, but rather decreased.

**Conclusion:**

Our findings suggest that both bone resorption and bone formation were inhibited by long-term administration of Bis alone, whereas combination therapy with Bis + statin may be associated with a less marked inhibition of bone metabolism. Cardiovascular disease is highly prevalent in RA patients and some patients are prescribed statins and bisphosphonate. Bis + statin may confer more benefit to the bone metabolism of these patients compared to Bis alone.

## Introduction

Loss of bone mass is frequently seen in patients with rheumatoid arthritis (RA). The main causes of osteoporosis in patients with RA are reportedly steroid therapy, postmenopausal changes in hormone balance (postmenopausal osteoporosis), and disuse bone atrophy associated with periarticular impairment [1,2]. Conversely, bone and cartilage damage in RA results from an imbalance between synthesis and degradation caused by cellular and cytokine-mediated inflammation. Growing evidence demonstrates that the increased bone resorption in bone diseases such as osteoporosis and RA is linked to the facilitation of osteoclast differentiation and activation by inflammatory cytokines of TNF-α and IL-1 [3-5]. Osteoprotegerin (OPG), a secreted soluble decoy receptor with homology to the members of the TNF receptor family, binds to the receptor activator of NF-κB ligand (RANKL) and blocks interactions with the receptor activator of NF-κB [6,7]. An imbalance in this system may play a part in the skeletal complications of RA [8].

Bisphosphonates (Bis), a class of agents taken up by osteoclasts and macrophages to inhibit the activity of inflammatory cytokines, are thought to inhibit the inflammation induced by these cells. Etidronate, a non-amino Bis, is reportedly effective in relieving pain in patients suffering from steroid-induced osteoporosis, postmenopausal osteoporosis, and osteoarthritis. Likewise, the amino-bisphosphonate risedronate is reportedly effective as a treatment for decreased bone density and vertebral fracture in patients on corticosteroid therapy [9,10]. Our group previously examined whether intermittent cyclical etidronate inhibits bone resorption or inflammatory changes over two study periods of 1.5 years and 3 years, in osteoporotic patients with RA [11,12]. Etidronate also inhibited the production of mediators related to inflammation, pain, and angiogenesis, namely IL-6, prostaglandin E_2_, substance P, and vascular endothelial growth factor, in synovial cells of arthritic models [12].

Statins have recently been reported to stimulate bone formation *in vivo *[13,14] as effectively as vitamin K_2 _(vit K_2_), to stimulate osteoblastogenesis, and to inhibit osteoclastogenesis in human bone marrow cell culture [15], and also to inhibit bone loss induced by prednisolone (PSL) in rats [16]. Clinical trials by our group have clarified that Vit K_2 _alone or in combination with Bis may inhibit osteoclast induction by decreasing the levels of RANKL [17].

The present study examined the effects of Bis (etidronate, alendronate and risedronate) alone and in combination therapy with statins on bone mineral density (BMD) and on four markers of bone metabolism, namely N-terminal telopeptide of type I collagen (NTX), tartrate-resistant acid phosphate-5b (TRACP-5b), C-terminal propeptide of type I procollagen (PICP) and RANKL in patients with RA.

## Materials and methods

The study subjects were 77 patients with RA who fulfilled the diagnostic criteria of the American College of Rheumatology (ACR) [18] and had been receiving PSL and Bis for over 4 years. They were divided into two groups: Bis (*n *= 42) and Bis + statin (*n *= 35). The drugs administered in the Bis group were 35 mg of alendronate (*n *= 31 patients), 400 mg of etidronate (*n *= 4 patients) and 17.5 mg of risedronate (*n *= 7 patients). The drugs administered in the Bis + statin group were alendronate and 2 mg of pitavastatin (*n *= 26 patients), etidronate and pitavastatin (*n *= 5 patients), and risedronate and pitavastatin (*n *= 4 patients). The patients diagnosed with hyperlipidemia also received pitavastatin, with their informed consent. The patients were selected from the outpatient clinic of our department in Tokyo Metropolitan Bokutoh Hospital. A 400 mg dose of etidronate was administered orally between meals for 2 weeks, and was then withheld for the next 10 weeks. This 12-week period was defined as one cycle of etidronate treatment, and the cycle was repeated 6 times (72 weeks, 18 months).

The mean age ± SD of the patients was 66.4 ± 6.2 years in the Bis group and 65.3 ± 6.8 years in the Bis + statin group. The mean disease duration was 24.9 ± 10.6 years in the Bis group and 20.8 ± 8.9 years in the Bis + statin group. Adrenal corticosteroid in the form of 1-7 mg of PSL was administered once daily to 30 patients in the Bis group and 28 patients in the Bis + statin group (mean PSL doses of 2.4 ± 2.0 mg and 2.7 ± 2.1 mg, respectively). A 0.5 μg dose of vitamin D_3 _was administered once daily to seven patients in the Bis group and three patients in the Bis + statin group. Calcium was not administered to patients in either group. Almost all of the patients received disease-modifying anti-rheumatic drugs (DMARDs). The most frequently used DMARDs were methotrexate (MTX) and salazosulfapyridine, but eight patients (six Bis patients and two Bis + statin patients) were prescribed biological agents (TNF-α blockades). MTX was administered to 34 Bis and 25 Bis + statin patients, at mean MTX doses of 5.2 ± 3.2 mg (range 2 to 10 mg) and 4.1 ± 3.1 mg (range 2 to 10 mg), respectively. The duration of Bis treatment exceeded 5 years in 27 Bis patients and in 22 Bis + statin patients, averaging over 4.2 years and over 3.9 years, respectively. There was no period of drug cessation before the study was initiated. The PSL and MTX doses were not increased, and no other medications were administered. This study was approved by the Ethical Committee of Tokyo Metropolitan Bokutoh Hospital. All patients gave their informed consent. No significant differences between the Bis and Bis + statin groups were observed in the background data or in the baseline data on serum bone markers and BMD (Tables 1 and 2). During an 18-month period of treatment and follow-up, the BMD of the radius, lumbar spine (vertebra L3), and femoral neck were measured by dual-energy x-ray absorptiometry (DXA) (QDR4500, Hologic Inc., Bedford, MA, USA). The following markers were measured by ELISA and were compared in the two groups: serum NTX and TRACP-5b as markers of bone resorption (ELISA, Alere Medical, Tokyo, Japan; DS Pharma Biomedical, Osaka, Japan), serum PICP as a marker of osteogenesis (ELISA, Takara, Japan), and serum levels of RANKL (ELISA, Biomedica, Vienna, Austria) (Table 3).

**Table 1:** Background data for patients with rheumatoid arthritis (RA)

	Bisphosphonate group (*n *= 42)	Bisphosphonate + statin group (*n *= 35)	*P*-value (Student's *t*-test)
**Age, years**	66.4 ± 6.2 (49.3, 77.6)	65.3 ± 6.8 (52, 76.8)	0.463
**Sex**			
Male	2	0	
Female	40	35	
59 >	3	8	
60-69	24	17	
70 <	13	10	
**Disease duration, years**	24.9 ± 10.6 (6.1, 54.3)	20.8 ± 8.9 (5.9, 38.)	0.083
**Bisphosphonates**			
Alendronate	31	26	
Etidronate	4	5	
Risedronate	7	4	
**Administered**			
> 4 years	29	22	
1 to 4 years	13	13	
**Prednisolone, mg**	2.4 ± 2.0 (0, 6.0)	2.8 ± 2.1 (0, 7.0)	0.355
+	30	28	
-	12	7	
**Methotrexate, mg**	5.2 ± 3.2 (0, 10)	4.1 ± 3.1 (0, 10)	0.140
+	34	25	
-	8	10	
**Vitamin D_3_, μg**	0.5	0.5	
+	7	3	
-	35	32	

Results are presented as mean ± SD (range) or number of patients. *P*-values are for comparisons between the two treatment groups.

**Table 2:** Baseline data for bone mineral density (BMD) and serum bone markers in patients with rheumatoid arthritis (RA)

BMD/serum bone markers	Bisphosphonate group (*n *= 42)	Bisphosphonate + statin group (*n *= 35)	*P*-value Student's *t*-test
**BMD, g/cm^2^**			
Radius	0.35 ± 0.08 (0.12, 0.54)	0.33 ± 0.13 (0.11, 0.63)	0.547
Lumbar spine	0.82 ± 0.18 (0.50, 1.32)	0.82 ± 0.180 (0.51, 1.40)	0.974
Femoral neck	0.64 ± 0.09 (0.39, 0.81)	0.60 ± 0.13 (0.33, 0.79)	0.096
**NTX, nmol BCE/l**	16.3 ± 5.4 (9.7, 35.6)	15.8 ± 4.2 (9.3, 27.9)	0.690
**TRACP-5b, μU/dl**	357 ± 1287(131, 628)	350 ± 102 (174, 652)	0.820
**PICP, ng/ml**	612 ± 218 (384, 1490)	562 ± 205 (289, 1340)	0.315
**RANKL, pmol/l**	0.27 ± 0.36 (0.13, 1.92)	0.16 ± 0.11 (0.13, 0.63)	0.093

Results are presented as mean ± SD (range). NTX, N-terminal telopeptide of type I collagen; TRACP-5b, tartrate-resistant acid phosphate-5b; PICP, C-terminal propeptide of type I procollagen; RANKL, receptor activator of NF-κB ligand. *P*-values are for comparisons between the two treatment groups.

**Table 3:** Measurement methods

Measurement	Measurement method
BMD	DXA (QDR4500, Hologic Inc., Bedford. MA)
NTX TRACP-5b	ELISA (Alere Medical, Japan) ELISA (DS Pharma Biomedical, Japan)
PICP	ELISA (Takara, Japan)
RANKL	ELISA (Biomedica, Vienna, Austria)

BMD, bone mineral density; NTX, N-terminal telopeptide of type I collagen; TRACP-5b, tartrate-resistant acid phosphate-5b; PICP, C-terminal propeptide of type I procollagen; RANKL, receptor activator of NF-κB ligand; DXA, dual-energy x-ray absorptiometry; ELISA, enzyme-linked immunosorbent assay.

All data are expressed as means ± SD. All background data in the patients with RA were analyzed using Student's *t*-test. Percentage changes in all parameters (BMD measured by DXA, NTX, TRACP-5b, PICP, and RANKL) at 0, 6, 12, and 18 months in the Bis and Bis + statin groups relative to the baseline measurements were evaluated using the Wilcoxon signed ranks test, and differences in percentage changes at 0, 6, 12, and 18 months between the Bis and Bis + statin groups were evaluated using the Mann-Whitney *U*-test. A *P*-value less than 0.05 was assumed to indicate a statistically significant difference.

## Results

### Measurement of BMD by DXA

**Figure 1:**
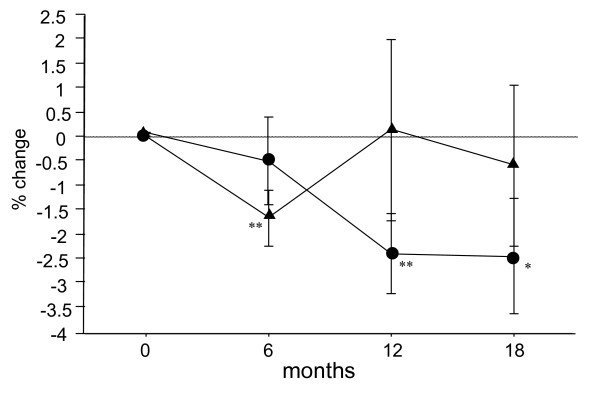
**Percentage changes in bone mineral density (BMD) of the radius measured by dual-energy x-ray absorptiometry (DXA) at 6, 12 and 18 months after the start of treatment in the Bisphosphonate (Bis) and Bis + statin groups**. Circles represent the Bis group and triangles represent the Bis + statin group. Significant decreases in BMD of the radius were observed at 12 and 18 months in the Bis group. In the Bis + statin group, a significant decrease was only observed at 6 months, and after 6 months tended to increase at 12 and 18 months. *P < 0.05, **P < 0.01, for comparison to baseline for each group.

Background data on the patients with RA are shown in Table 1. No significant differences in these parameters were observed between the two groups. The percent changes in BMD of the radius were -0.6% and -1.7% at 6 months after the start of treatment, -2.5% and 0.1% at 12 months and -2.5% and -0.6% at 18 months in the Bis and Bis + statin groups respectively. Statistical analysis of these two groups at 12 and 18 months showed significant decreases in the Bis group and increases at a sub-significant level in the Bis + statin group (Figure 1).

**Figure 2:**
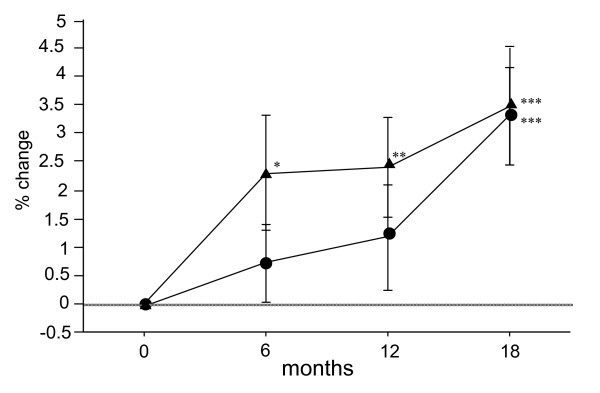
**Percentage changes in bone mineral density (BMD) of the lumbar spine measured by dual-energy x-ray absorptiometry (DXA) at 6, 12 and 18 months after the start of treatment in the bisphosphonate (Bis) and Bis + statin groups**. Circles represent the Bis group and triangles represent the Bis + statin group. Significant increases in BMD of the lumbar spine were observed at 6, 12 and 18 months in the Bis + statin group, but in the Bis group a significant increase was only observed at 18 months. *P < 0.05, **P < 0.01, ***P < 0.001, for comparison to baseline for each group.

The percent changes in BMD of the lumbar spine in the Bis and Bis + statin groups were 0.7% and 2.3% at 6 months after the start of treatment, 1.2% and 2.4% at 12 months, and 3.3% and 3.5% at 18 months, respectively. Statistical analysis of these two groups showed significant increases at 6, 12, and 18 months in the Bis + statin group and a significant increase at 18 months in the Bis group (Figure 2).

**Figure 3:**
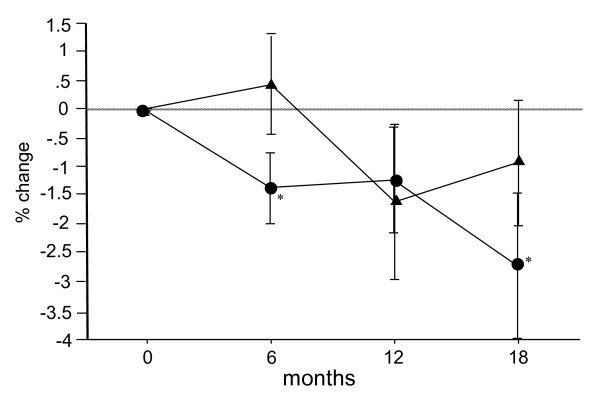
**Percentage changes in bone mineral density (BMD) of the femoral neck measured by dual-energy x-ray absorptiometry (DXA) at 6, 12 and 18 months after the start of treatment in the bisphosphonate (Bis) and Bis + statin groups**. Circles represent the Bis group and triangles represent the Bis + statin group. Significant decreases in BMD of the femoral neck were observed at 6 and 18 months in the Bis group, but in the Bis + statin group a significant decrease in BMD was not observed during the follow-up period. *P < 0.05, **P < 0.01, for comparison to baseline for each group.

The percent changes in BMD of the femoral neck in the Bis and Bis + statin groups were -1.3% and 0.4% at 6 months after the start of treatment, -1.2% and -1.6% at 12 months, and -2.7% and -0.9% at 18 months, respectively. Statistical analysis of these two groups over the follow-up period showed significant decreases in the Bis group at 6 and 18 months, and no significant decreases in the Bis + statin group (Figure 3).

These results demonstrated a significant increase in BMD of the lumbar spine during the follow-up period in the Bis + statin group. The BMD of the radius and femoral neck decreased significantly during the follow-up period in the Bis group, but not in the Bis + statin group.

### Measurement of serum NTX, TRACP-5b, PICP and RANKL as markers of bone resorption, formation, and destruction

**Figure 4:**
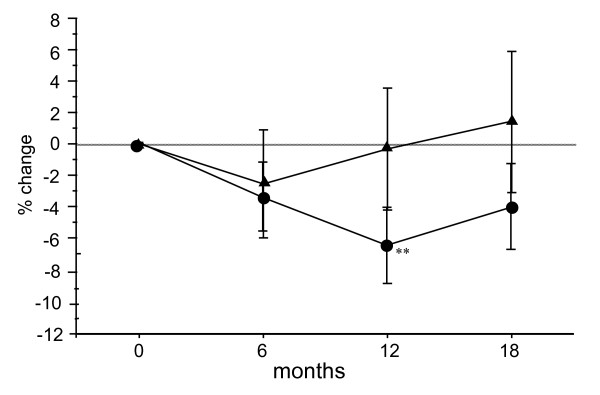
**Percentage changes in serum N-terminal telopeptide of type I collagen (NTX) at 6, 12 and 18 months after the start of treatment in the bisphosphonate (Bis) and Bis + statin groups**. Circles represent the Bis group and triangles represent the Bis + statin group. A significant decrease in serum NTX was observed at 12 months in the Bis group, but in the Bis + statin group, serum NTX was up-regulated during the follow-up period except at 6 months. *P < 0.05, **P < 0.01, for comparison to baseline for each group.

**Figure 5:**
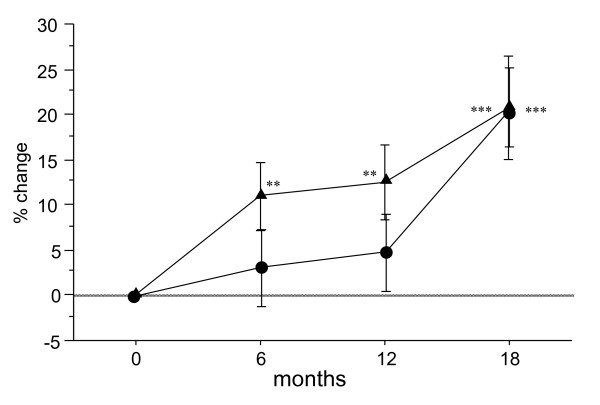
**Percentage changes in serum tartrate-resistant acid phosphate-5b (TRACP-5b) at 6, 12 and 18 months after the start of treatment in the bisphosphonate (Bis) and Bis + statin groups**. Circles represent the Bis group and triangles represent the Bis + statin group. Significant increases in serum TRACP-5b were observed at 6, 12 and 18 months in the Bis + statin group, but in the Bis group a significant increase was only observed at 18 months. *P < 0.05, *P < 0.01, ***P < 0.001, for comparison to baseline for each group.

The percent changes in serum NTX in the Bis and Bis + statin groups were -3.8% and -2.6% at 6 months after the start of treatment, -7.2% and -0.3% at 12 months, and -4.5% and 1.4% at 18 months, respectively (Figure 4). Serum NTX at 12 months was significantly decreased in the Bis group. The Bis + statin group exhibited no such decrease at 12 months, but rather an up-regulation in the level of serum NTX after 6 months during the follow-up period. Namely, inhibition of bone resorption was gradually restricted after the start of Bis + statin treatment. In addition to NTX, the percent changes in serum TRACP-5b, a marker of bone resorption, were 3.2% and 10.9% at 6 months after the start of treatment, 4.9% and 12.5% at 12 months, and 20.8% and 20.7% at 18 months, respectively, in the Bis and Bis + statin groups (Figure 5). Serum TRACP-5b was significantly increased throughout the whole follow-up period in the Bis + statin group, but only at 18 months in the Bis group.

**Figure 6:**
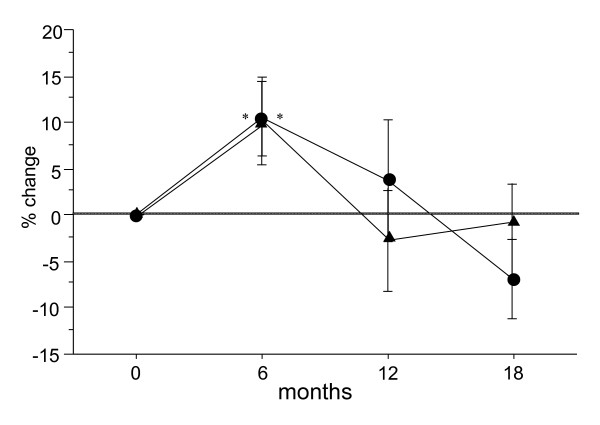
**Percentage changes in serum C- terminal propeptide of type I procollagen (PICP) at 6, 12 and 18 months after the start of treatment in the bisphosphonate (Bis) and Bis + statin groups**. A significant increase in serum PICP was observed at 6 months in the Bis and Bis + statin group. On the other hand, in the Bis + statin group serum PICP tends to increase after 12 months. *P < 0.05, **P < 0.01, for comparison to baseline for each group.

The percent changes in serum PICP in the Bis and Bis + statin groups were 10.6% and 10.0% at 6 months after the start of treatment, 3.7% and -2.9% at 12 months, and -7.0% and -1.5% at 18 months, respectively (Figure 6). A significant increase in serum PICP, a marker of bone formation, was observed at 6 months in both groups. Serum PICP gradually decreased after 6 months in the Bis and Bis + statin group but gradually recovered to baseline in the Bis + statin group. In contrast, serum PICP did not observably recover, but rather decreased in the Bis group.

The serum RANKL levels tended to decrease during the follow-up period in the Bis and Bis + statin groups. The serum RANKL had decreased to below a detectable level over more than 4 years of treatment with Bis (data not shown).

## Discussion

In this study we investigated the effects of Bis, alone or in combination with statins, on bone resorption and bone formation based on BMD and various bone markers in patients with RA who had been receiving Bis for more than 4 years. Alendronate prevents the inhibition of osteoclast formation, bone resorption, and kinase activation *in vitro *via inhibition of metabolism from farnesyl disphosphate to geranylgeranyl diphosphate in the mevalonate pathway [19]. Hydroxymethylglutanyl-CoA (HMG-CoA) reductase inhibitors such as lovastatin, pravastatin, and simvastatin also block the conversion of HMG-CoA to mevalonate, and inhibit the production of farnesyl-PP and geranylgeranyl-PP. Meanwhile, amino-bisphosphonates and HMG-CoA reductase inhibitors have been shown to increase macrophage (osteoclast-like cell) apoptosis [20,21]. Statins and Bis inhibit different enzymes within the same pathway. As such, we can expect statins and Bis to confer similar effects on osteoclasts and macrophages.

HMG-CoA reductase inhibitors are also known to increase new bone formation from osteoblasts and to accelerate the promoter activity of bone morphorogic protein-2 (BMP-2), a member of the BMP family [13]. Sugiyama *et al. *reported that simvastatin, but not pravastatin, induced BMP-2 in human osteosarcoma cells [22]. The statin-mediated activation of BMP-2 promoter was completely inhibited by the downstream metabolite of HMG-CoA reductase, mevalonate, which meant that the activation was a result of the inhibition of the enzyme. In both prospective and retrospective studies of the same patients, BMD of the lumbar spine in patients administered fluvastatin was significantly increased compared with administration of pravastatin at 6 and 12 months after the start of the treatment [23]. In a similar study of female patients, HMG-CoA reductase inhibitors significantly increased BMD of the femoral neck after 15 months [24].

In our comparison between the Bis group and Bis + statin groups, the Bis group had a small increase in BMD at the lumbar spine, versus significant decreases in BMD at the radius and femoral neck during the follow-up period. In the Bis + statin group, the BMD of the lumbar spine significantly increased after 6 months of follow-up, while the BMD of the radius and femoral neck did not decrease significantly. Compared with subjects administered Bis alone, the subjects receiving combination therapy had a significant increase in BMD at the lumbar spine after the start of treatment, while the BMD of the radius and femoral neck stayed at baseline levels.

Several studies [25-27] have documented reductions in NTX and increased BMD in postmenopausal osteoporotic women administered bisphosphonates. The production of NTX, a marker of bone resorption, decreased during the follow-up period in the Bis group while remaining unchanged following commencement of statin treatment in the Bis + statin group. These results indicate that bone resorption was continuously inhibited in the Bis group but was not inhibited in the Bis + statin group.

Serum TRACP-5b, a marker of bone resorption, increased during the follow-up period in both of our groups and significantly increased after the start of statin treatment in the Bis + statin group. Serum TRACP-5b and TRACP protein have been found to increase significantly in RA and conditions related to increased bone resorption [28]. The increased total TRACP-5 protein in RA was not derived from the osteoclasts, but may have been a secreted product of inflammatory macrophages and dendritic cells [29].

In our results, serum RANKL remained below detectable levels in our patients who received Bis over a long period, hence osteoclast differentiation via the RANK/RANKL system may have been depressed. The increase of TRACP-5b may thus have been linked to osteoclast differentiation or activation via TNF-αor IL-1, and macrophages or dendritic cells. TRACP-5b increased after the start of treatment in both groups, whereas NTX increased after 6 months only in the Bis + statin group. These results show that statins may depress the production of NTX rather than the production of TRACP-5b. Janckila *et al. *found no significant correlation between TRACP-5b and serum bone alkaline phosphatase (BAP) or NTX [30] in RA and observed that serum TRACP-5b decreased less in response to clodronate than in response to urinary NTX [31].

Patients with RA experience systemic osteoporosis and produce various inflammatory cytokines that accelerate bone resorption in joints, and high concentrations of soluble RANKL are detected in their synovial fluid [32]. Serum RANKL was already quite low at the start of treatment in both of our groups, and it decreased further throughout the follow-up period. Some studies have documented that Bis alone or in combination therapy reduces RANKL [17,33,34]. We have already reported that vit K_2 _alone or Bis + vit K_2 _reduces RANKL to below the detectable level at 6 months after the start of treatment [17].

Our measurement of the bone formation marker serum PICP, showed a temporal increase in both groups at 6 months. At 12 months the increase was not maintained in either group, and by 18 months, PICP had gradually returned to baseline in the Bis + statin groups. When Mostaza *et al. *[35] administered pravastatin to hypercholesterolemic postmenopausal women, the agent increased PINP, a marker of bone formation, without affecting bone resorption. When alendronate was administered alone and in combination with parathyroid hormone (PTH), a marked increase of bone formation was observed in the PTH alone group, but not in the combination group, and bone resorption decreased in both groups [36]. These results suggest that the concurrent use of alendronate may reduce the anabolic effects of PTH.

Bis + statin appeared to block the inhibition of bone resorption by up-regulating the levels of NTX and TRCP-5b, whereas the level of RANKL was inhibited in both groups. In our measurement of BMD in the Bis + statin subjects, BMD of the radius and femoral neck stayed at baseline levels despite temporal decreases, while BMD of the lumbar spine increased significantly.

## Conclusions

Our findings suggest that both bone resorption and bone formation were inhibited by long-term administration of Bis alone, whereas combination therapy with Bis + statin may be associated with a less marked inhibition of bone metabolism. We would like to recommend a combination therapy of bisphosphonate with statin or vit K_2_, to promote bone formation for the treatment or prevention of osteoporosis in RA patients.

## Abbreviations

Bis: bisphosphonates; BMD: bone mineral density; BMP: bone morphorogic protein; DMARD: disease-modifying anti-rheumatic drug; DXA: dual-energy x-ray absorptiometry; ELISA: enzyme-linked immunosorbent assay; HMG-CoA: hydroxymethylglutanyl-CoA; MTX: methotrexate; NTX: N-terminal telopeptide of type I collagen; OPG: osteoprotogerin; PTH: parathyroid hormone; TRACP-5b: tartrate-resistant acid phosphate-5b; PICP: C- terminal propeptide of type I procollagen; RANKL: receptor activator of NF-κB ligand; PSL: prednisolone; RA: rheumatoid arthritis; TNF: tumor necrosis factor; vit K2: vitamin K_2_.

## Competing interests

The authors declare that they have no competing interests.

## Authors' contributions

All authors contributed to the final manuscript. MN participated in the design of the study, statistical analysis and interpretation of the data, drafting the article and critical revision of the article for important intellectual content. HT and KW assisted with collection and acquisition of the data and critically revised the manuscript. KS and YN assisted with critical revision of the manuscript. All authors have read and approved the manuscript for publication.
